# *N*-Butyrylated Hyaluronic Acid Achieves Anti-Inflammatory Effects In Vitro and in Adjuvant-Induced Immune Activation in Rats

**DOI:** 10.3390/molecules27103267

**Published:** 2022-05-19

**Authors:** Xue Luan, Zhongcheng Cong, Tassos P. Anastassiades, Yin Gao

**Affiliations:** 1 Key Laboratory for Molecular Enzymology and Engineering of Ministry of Education, School of Life Sciences, Jilin University, Changchun 130012, China; luanxue19@mails.jlu.edu.cn (X.L.); congzhongcheng@luye.com (Z.C.); 2 Division of Rheumatology, Department of Medicine, Queen’s University, Kingston, ON K7L 4B4, Canada; anastass@queensu.ca

**Keywords:** *N*-butyrylation, anti-inflammation, antioxidation, NF-κB/MAPK, JAK-STAT

## Abstract

Previously synthesized *N*-butyrylated hyaluronic acid (BHA) provides anti-inflammatory effects in rat models of acute gouty arthritis and hyperuricemia. However, the mechanism of action remains to be elucidated. Herein, the anti-inflammatory and antioxidative activities of BHA and the targeted signaling pathways were explored with LPS-induced RAW264.7 and an adjuvant-induced inflammation in a rat model. Results indicated that BHA inhibited the generation of pro-inflammatory cytokines TNFα, IL-1β and IL-6, reduced ROS production and down-regulated JAK1-STAT1/3 signaling pathways in LPS-induced RAW264.7. In vivo, BHA alleviated paw and joint swelling, decreased inflammatory cell infiltration in paw tissues, suppressed gene expressions of p38 and p65, down-regulated the NF-κB and MAPK signaling pathways and reduced protein levels of TNFα, IL-1β and IL-6 in joint tissues of arthritis rats. This study demonstrated the pivotal role of BHA in anti-inflammation and anti-oxidation, suggesting the potential clinical value of BHA in the prevention of inflammatory arthritis and is worthy for development as a new pharmacological treatment.

## 1. Introduction

Chronic joint inflammation with symptoms including joint pain, swelling, cartilage destruction and bone erosion is a major cause of disability [1]. Although numerous anti-inflammatory agents have been developed, the response at the inflammation site to these drugs remains unsatisfactory due to the complexity of unrevealed pathological mechanisms. In particular, the inhibition of one target may not be sufficient to halt or reverse the progression of the disease. Under the influence of inflammatory mediators, the concentrations of reactive oxygen species (ROS) are elevated in the inflamed tissues [2]. Both ROS and the pro-inflammatory factors have been reported to trigger nuclear factor kappa-B (NF-κB) and mitogen-activated protein kinase (MAPK) signaling pathways [3,4,5]. This process leads to the further production of pro-inflammatory cytokines and induces the production of metalloproteinases and adhesion molecules that eventually regulate immune cells, synoviocytes and chondrocytes, causing cartilage to undergo progressive degradation and ultimately results in joint-space narrowing and joint destruction [4,5].

Currently, non-steroidal anti-inflammatory drugs (NSAIDs) and corticosteroids are used as the common treatment to relieve joint pain and swelling associated with chronic joint inflammation [6,7]. However, the adverse side effects of these drugs toward the cardiovascular and gastrointestinal systems limit their suitability as long-term treatments for chronic inflammation (i.e., Rheumatoid arthritis, RA) [8]. In addition, a significant proportion of patients did not achieve good clinical responses and even developed adverse reactions to disease-modifying antirheumatic drugs (DMARDs) [9]. Therefore, alternative anti-inflammatory methods are urgently required.

Hyaluronic acid (HA) is a naturally occurring polysaccharide that is composed of repeating N-acetyl-glucosamine and glucuronic acid (GlcUA) units that are linked together via alternating β-1,4- and β-1,3-glycosidic bonds [10]. HA serves as a major component of synovial fluid and extracellular matrices of cartilage, thus protecting articular cartilage from damage and playing a central role in joint lubrication [11]. It has been found that the concentration of HA in joints is decreased in elderly people compared to their younger counterparts [12]. The intra-articular administration of HA has been commonly used to treat osteoarthritis to improve joint pain, stiffness and function in patients [13,14]. However, treatment with HA has not inhibited disease progression, and its anti-inflammatory effect was augmented [15]. Therefore, the development of HA derivatives with anti-inflammatory activities can provide a new strategy for joint protection.

It is reported that the molecular weight of HA is significantly reduced in patients with RA as well as those with other inflammatory joint diseases in comparison to healthy people [12,16]. HA undergoes fragmentation in the inflammation site and stimulates a pro-inflammatory cellular response [15,17]. We have introduced a structurally modified *N*-butyrylated HA (BHA), which can inhibit the production of pro-inflammatory cytokines in low molecular-weight HA stimulated human macrophages [18]. Moreover, BHA reduced the level of inflammatory factors (IL-1β, IL-8, MCP-1 and IFN-γ) in the serum of gouty arthritis rats and reduced the serum uric acid concentration and ROS level in mice with hyperuricemia [19], showing therapeutic effects against acute gout in animal models. Anti-inflammatory and antioxidant therapies were shown to arrest bone damage in RA patients [3,20,21]. In this regard, the antioxidant and anti-inflammatory effects of BHA were evaluated in vitro with LPS-induced RAW264.7 mouse macrophage and in vivo with an adjuvant-induced local inflammation in rat models. BHA inhibited the production of ROS and inflammatory factors and suppressed inflammation-associated signaling pathways, suggesting that BHA can provide an effective method for treating joint inflammation.

## 2. Results

### 2.1. Characterization of Synthesized BHA

**Figure 1:**
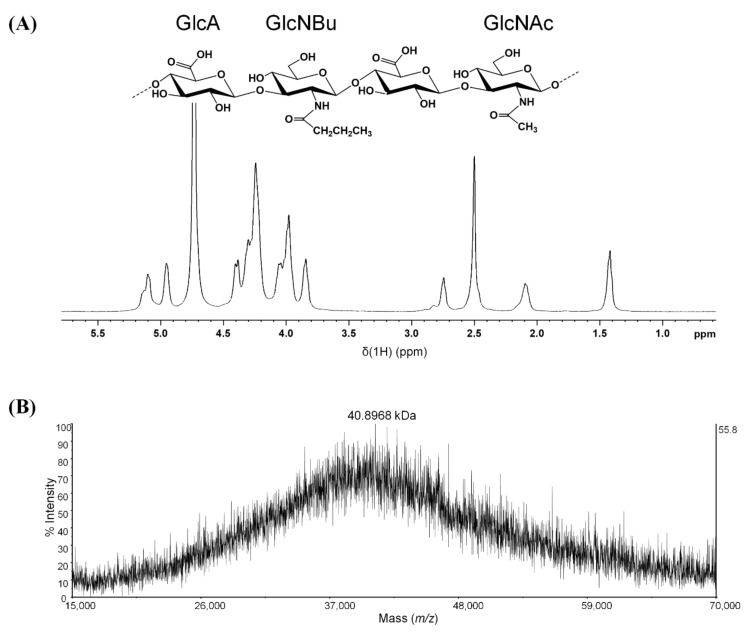
^1^H NMR and MALDI-TOF analysis of synthesized BHA. (**A**) The structure and the ^1^H NMR spectrum of BHA. Prior to characterization, BHA was dissolved in D_2_O as a concentration of 10 mg/mL. ^1^H NMR spectra of BHA were recorded at 348 K with a 500 MHz NMR spectrometer. (**B**) The mass spectrum of BHA. BHA was prepared in 2,5-dihydroxybenzoic acid with an equal volume of matrix solution. BHA: partially butylated HA; GlcA: D-glucuronic acid; GlcNAc: *N*-acetyl-D-glucosamine; GlcNBu: *N*-butyl-D-glucosamine.

The spectrum of BHA in Figure 1A exhibited both -CH_2_CH_2_CH_3_ and -CH_3_ proton signals, and the butylation-to-acetylation ratio of the sample of BHA was determined to be 34.2 ± 0.2% based on the integration ratio of methyl protons in the -CH_2_CH_2_CH_3_ moiety to the total methyl protons in the GlcNAc and -CH_2_CH_2_CH_3_ moieties. MALDI-TOF was shown to produce results with reasonably accurate molecular weights [22,23]. The molecular weights of BHA were measured to be approximately 41 kDa (Figure 1B). This synthesized BHA was used in all subsequent cellular and animal experiments.

### 2.2. Effects of BHA on Cell Proliferation

**Figure 2:**
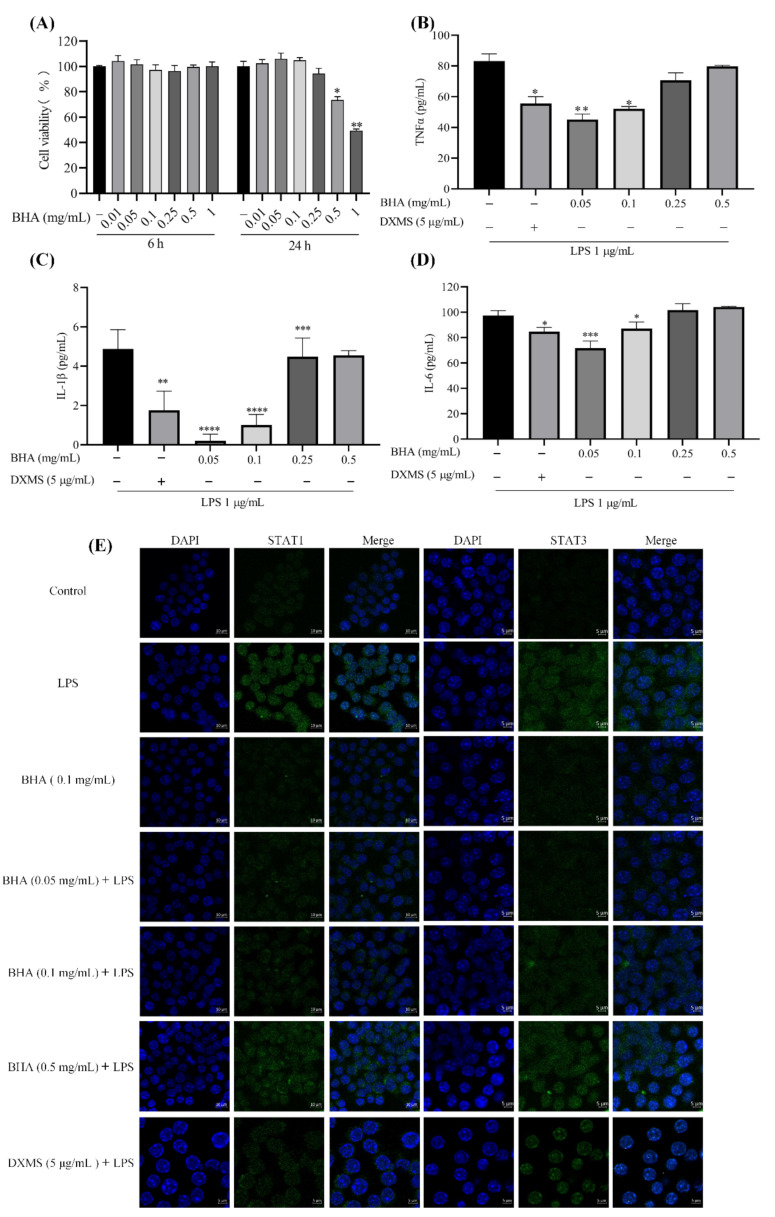
(**A**) Effects of BHA on the viability of RAW264.7 cells. Cells were incubated with BHA (0.01, 0.05, 0.1, 0.25, 0.5 or 1 mg/mL) for 6 and 24 h. Cell viability was measured using MTT assays. Data are presented as the mean ± SEM of three independent experiments. (**B**–**D**) Validation of the anti-inflammatory effects of BHA on LPS-induced RAW264.7 macrophage cells. Cells were pre-treated with various concentrations of BHA for 6 h and stimulated with 1 µg/mL of LPS for 12 h. Supernatants were collected after LPS stimulation, and the protein levels of TNFα, IL-1β and IL-6 were quantified by corresponding ELISA kits. Data are presented as the mean ± SEM of three independent experiments. (**E**) The expressions of STAT1 and STAT3 were detected by confocal laser microscopy. Cells were fixed, permeabilized and then incubated with anti-STAT1 and STAT3 antibodies followed by the incubation with FITC conjugated anti-rabbit IgG antibody (green). The nuclei were stained with DAPI (blue). Scale bar = 5 µm. One-way ANOVA was used to compare the results between each group. * *p* < 0.05, ** *p* *<* 0.01, *** *p < * 0.001, **** *p < * 0.0001. ^*^ Compared with the untreated LPS-induced model group.

As shown in Figure 2A, upon 6 h of treatment with BHA at a concentration range from 0.05 to 1 mg/mL, no cytotoxic effects were observed. After 24 h of incubation, BHA exhibited no obvious cytotoxic effects in the concentration range from 0.05 to 0.25 mg/mL, while higher concentrations of 0.5 and 1 mg/mL exhibited various degrees of cytotoxicity.

### 2.3. Effects of BHA on the Expression of Inflammation Factors in LPS-Induced RAW264.7 Marcrophage

The expression of TNFα was not affected by BHA at a concentration of 0.01 mg/mL (Supplementary Materials Figure S1). Thus, BHA concentrations of 0.05, 0.1, 0.25 and 0.5 mg/mL were selected for the subsequent cellular experiments. As shown in Figure 2B–D, BHA at lower concentrations (0.05 and 0.1 mg/mL) significantly suppressed the expressions of TNFα (*p* < 0.05), IL-1β (*p* < 0.0001) and IL-6 (*p* < 0.05) in LPS-stimulated RAW264.7 cells. Moreover, BHA at lower concentrations provided greater anti-inflammatory efficacy than were achieved at higher concentrations (0.25 and 0.5 mg/mL).

### 2.4. BHA Inhibits LPS-Induced STAT1 and STAT3 Expression

LPS-treated cells showed increased expressions of STAT1 and STAT3 (Figure 2E). DXMS and BHA (0.05 and 0.1 mg/mL) pretreatments dramatically reduced LPS-induced nucleocytoplasmic translocation of STAT1 and STAT3. On the other hand, BHA at the concentration of 0.5 mg/mL did not exhibit this effect, suggesting that the concentration of BHA is critical for providing anti-inflammatory effects.

### 2.5. Effects of BHA on Oxidative Stress

**Figure 3:**
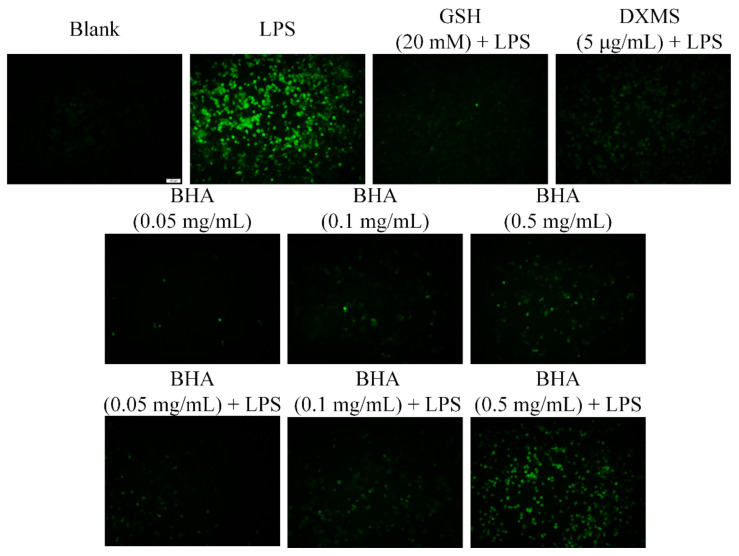
Evaluation of the antioxidant activity of BHA in LPS-induced RAW264.7 macrophages. Cells were treated with various concentrations of BHA for 12 h in the presence or absence of LPS (1 µg/mL) for 12 h. The generation of ROS was measured and confirmed via DCFH-DA assays.

LPS induction led to an elevation of the ROS levels in RAW264.7 cells (Figure 3). Treatment with DXMS, BHA (0.05 and 0.1 mg/mL) and the positive control reducing agent glutathione (GHS) reduced ROS production compared with that observed in the LPS-induced cells without treatments. BHA at a higher concentration (0.5 mg/mL) was not as effective as the lower concentrations in reducing ROS levels. Therefore, a low dosage of BHA was used in the in vivo experiments.

### 2.6. Therapeutic Effects of BHA on AIA Rats

**Figure 4:**
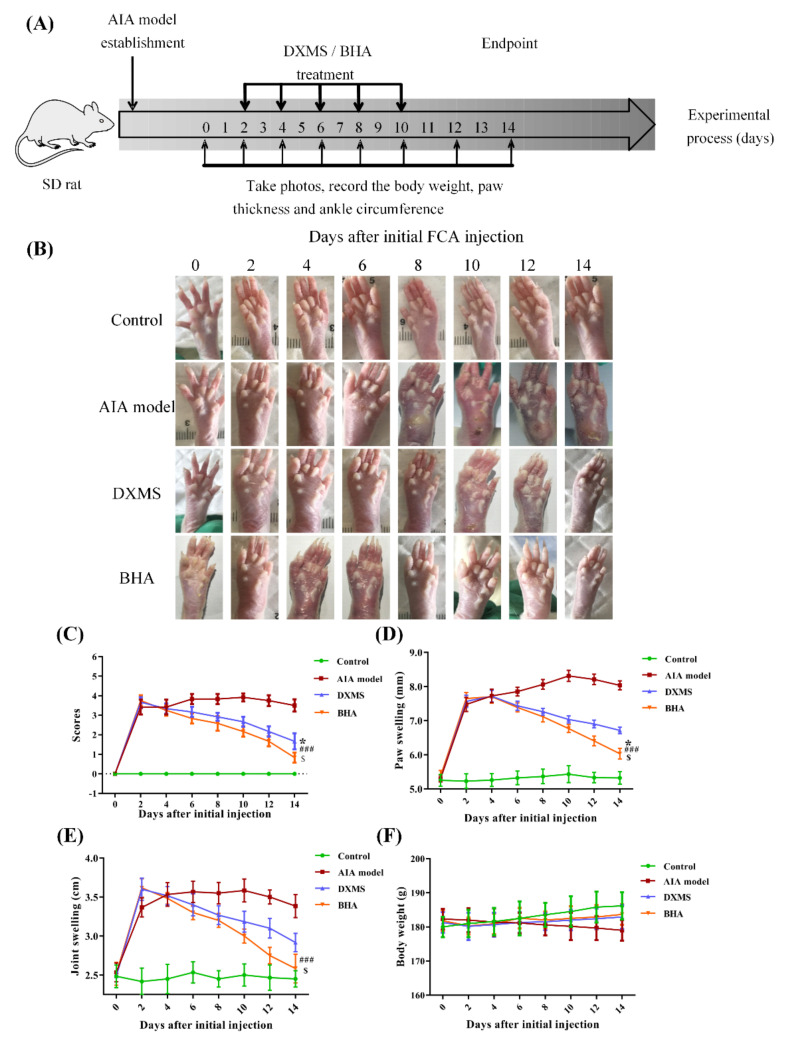
(**A**) Schematic illustration summarizing the treatment protocol that was used for the AIA rats. (**B**) Photographs of the rat paws taken at indicated time points for rats in different treatment groups during this therapeutic effectiveness study. (**C**–**F**) Evaluation of the therapeutic effects of BHA in AIA rats. (**C**) Arthritis scores, (**D**) paw swelling, (**E**) ankle swelling and (**F**) body weights were examined every 2 days. Values are shown as the mean ± SEM (*n* = 6 rats per group). * *p* < 0.05 was relative to the control group; ^###^ *p* < 0.001 was relative to the AIA model group; ^$^ *p* < 0.05 was relative to the DXMS treatment group.

In comparison to those in the control group, the paws of the FCA-treated rats exhibited increased redness, had more swelling and had higher temperatures on day 2 after FCA injection (Figure 4B). The subcutaneous injection of the positive control drug DXMS on days 2, 4, 6 and 8 significantly reduced the scores (*p* < 0.001) of ankle inflammation and bone erosion (Figure 4C) and also relieved paw (Figure 4D, *p* < 0.001) and joint swelling (Figure 4E, *p* < 0.001). The body weights of the rats remained constant throughout the duration of the experiment (Figure 4F). Treatment with BHA provided a better effect than DXMS by reducing the scores and more effectively relieved paw and joint swelling. The paws of the rats subjected to the BHA treatment showed an obvious recovery to normal status at day 14 (Figure 4B); thus, day 14 was set as the end point of the experiments.

**Figure 5:**
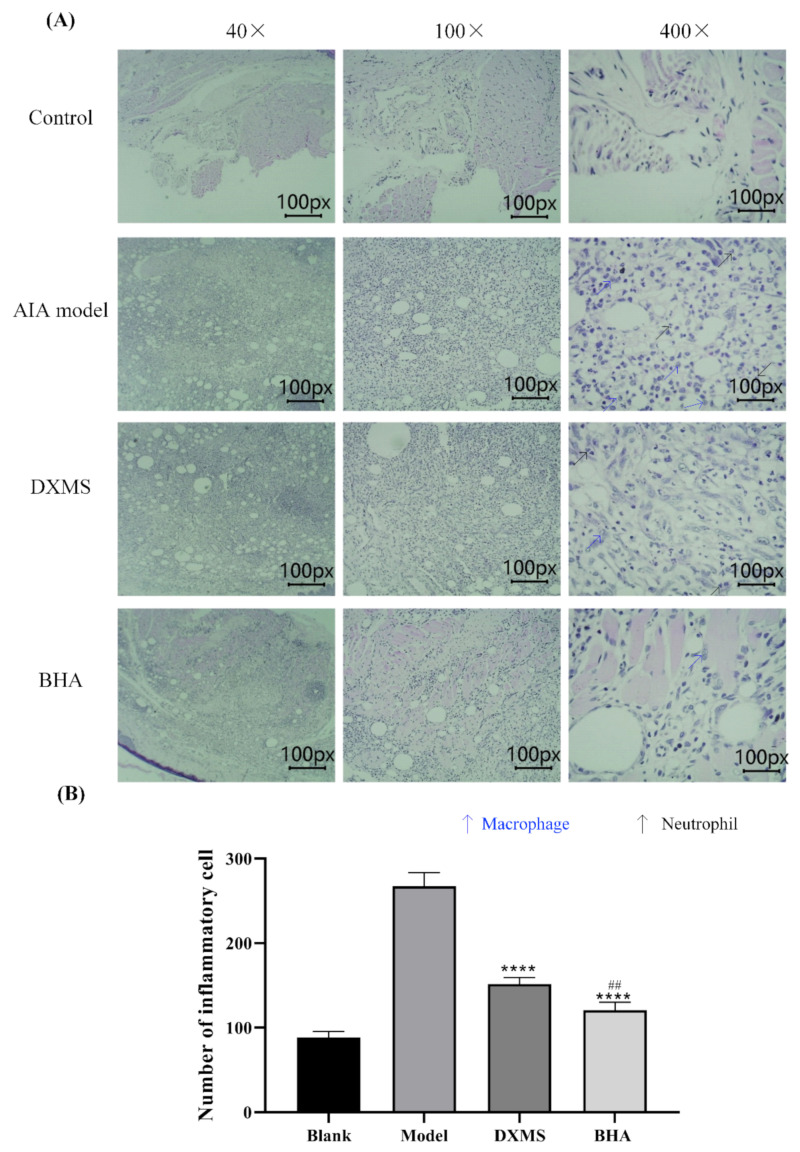
(**A**) Histopathological assessment of the paw tissues of rats. The rats were sacrificed at the endpoints of these experiments. The paw tissues of these rats were then excised and fixed in 4% paraformaldehyde. The histological sections were stained with hematoxylin and eosin. Microscopy at magnifications of 40×, 100× and 400× are shown for typical areas for each of the five groups. The control group possessed normal paw tissue. Increased inflammatory cell infiltration was observed in the paw tissue of FCA-injected AIA rats in the model group. Treatment with DXMS and BHA partially prevented inflammation in the paw tissues of AIA rats. The black arrow indicates neutrophile, and the blue arrow indicates macrophage. (**B**) Quantification of the pro-inflammatory cells. Data are expressed as mean ± SEM (*n* = 5), **** *p* < 0.0001 relative to the model group; ^##^ *p* < 0.01 was relative to the DXMS treatment group.

Histopathological analysis demonstrated that the subcutaneous injection of FCA caused increased inflammatory cell infiltration in the paw tissues compared to the control group (Figure 5A). The administration of DXMS reduced the number of infiltrating inflammatory cells. Consistently, treatment with BHA significantly reduced the infiltration of pro-inflammatory cells (*p* < 0.0001) and provided a better attenuation of the inflammation reaction than DXMS (Figure 5B, *p* < 0.01).

### 2.7. Effects of BHA on the Expression of Pro-Inflammatory Factors in AIA Rats

**Figure 6:**
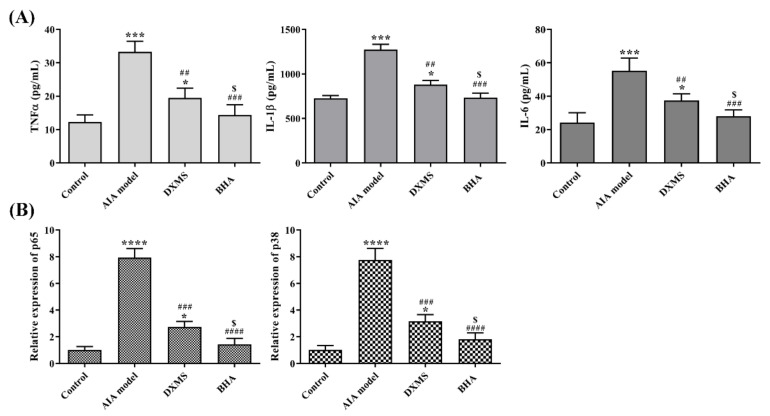
(**A**) The effects of BHA on cytokine levels in the inflammatory joint tissues in AIA rats. At the endpoint, the rats were sacrificed, and the protein levels of TNF-α, IL-1β and IL-6 in the ankle joint tissues were determined via ELISA assays. (**B**) The relative mRNA expression of inflammatory factors in the joint tissues obtained from AIA rats. The rats were euthanized at the endpoint of this experiment, and the expressions of p38 and p65 in ankle joint tissues were assayed by quantitative PCR. Values are shown as the mean ± SEM. * *p* < 0.05, *** *p* < 0.001 and **** *p* < 0.0001 were relative to the control group; ^##^ *p* < 0.01, ^###^ *p* < 0.001 and ^####^ *p* < 0.0001 were relative to the AIA model group; ^$^ *p* < 0.05 were relative to the DXMS treatment group.

The injection of FCA caused a substantial increase in the production of TNFα, IL-1β and IL-6 pro-inflammatory cytokines in the joint tissues of AIA rats in comparison to the control group (Figure 6A). DXMS treatment actively reduced the levels of the three inflammation-associated factors (TNFα, IL-1β and IL-6) in AIA rats by a significant degree (*p* < 0.01). The administration of BHA provided a stronger suppression of the production of these pro-inflammatory cytokines than was achieved by DXMS (*p* < 0.05).

### 2.8. Effects of BHA on the Regulation of NF-κB and MAPK Signaling Pathways

The overproduction of TNFα stimulates the activation of NF-κB and MAPK signaling events, thus leading to the production of IL-6 and IL-1β in inflamed tissue [24]. As shown in Figure 6B, the injection of FCA dramatically elevated the expression of p38 and p65 in the joint tissue of AIA rats compared to the control groups (*p* < 0.0001). Meanwhile, treatment with DXMS significantly down-regulated the expressions of p38 and p65 (*p* < 0.001). Consistently, BHA treatment provided a stronger attenuation of p38 and p65 expressions (*p* < 0.05).

## 3. Discussion

**Figure 7:**
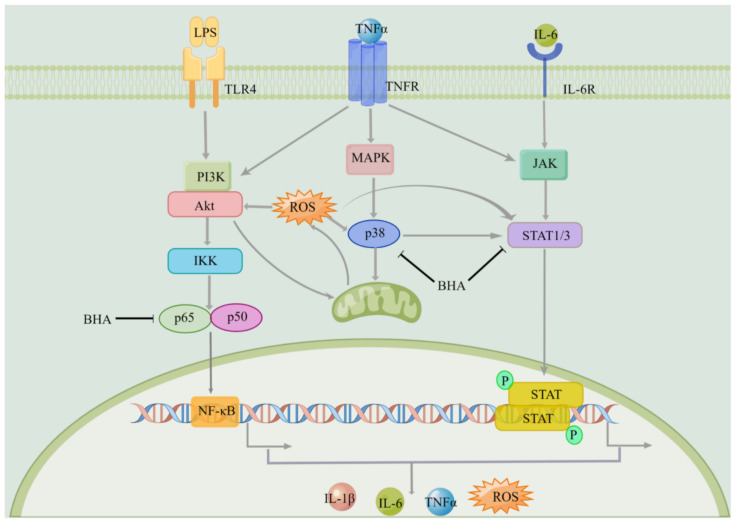
Anti-inflammatory effects of BHA and the mechanism of action. BHA interact with TLR4, interfering the expression of p65, p38 and STAT1/3, reducing the level of ROS and the production of cytokines TNFα, IL-1β and IL-6.

An activated macrophage is the primary source for the production of inflammatory cytokines [25], and it is involved in various inflammatory diseases [26]. In the current study, LPS-induced mouse macrophage was used as a model to evaluate the therapeutic potential of BHA. DXMS was used as a positive control drug [27]. In the current study, the maximum possible concentration of DXMS was used without inducing cellular toxic effects [28], and its effectiveness was compared with BHA. BHA (0.05 and 0.1 mg/mL) reduced the expression of STAT1/3 and pro-inflammatory cytokines (TNFα, IL-1β and IL-6) by statistically significant degrees in LPS-activated RAW264.7 mouse macrophages, showing the effect of BHA in regulating the JAK-STAT pathway (Figure 7). Furthermore, oxidative stress occurs as a consequence of inflammation and also enhances inflammation reactions [2]. The current study showed that BHA reduced ROS levels in LPS-induced macrophage. The results are consistent with previous studies that BHA inhibited the xanthine oxidase activity by regulating oxidative stress in oteracil potassium, and yeast extract powder induced hyperuricemia in mice model [19]. It is known that antioxidants can interfere with the activation of NF-κB signaling [29]. Reducing the ROS level can attenuate the inflammation reaction and inhibit the production of pro-inflammatory cytokines [29]. Therefore, BHA that combined both antioxidation and anti-inflammatory activities can be a more effective strategy for treating inflammation. However, the above studies showed that BHAs at higher concentrations lost its anti-inflammatory effects. Similarly, it has been reported that the therapeutic function of HA is dependent on its molecular weight and concentration [14,15]. Unfortunately, this dependency leads to problems if the exogenous HA undergoes fragmentation in the inflammation site, which can stimulate a pro-inflammatory cellular response [17,30,31]. We previously showed that a low molecular weight HA (AHA) can increase the expression levels of IL-1β in human macrophages [18]. Herein, we showed that AHA did not show antioxidant effects as the ROS level in the LPS induced mouse macrophages was remained (Supplementary Materials Figure S2), which can lead to immune activation. Since BHA is *N*-butyrylated HA with the butylation-to-acetylation ratio of 34.2 ± 0.2%, the degradation of BHA may lead to the production of small fragments of HA, which could induce inflammatory reactions. In addition, similarly to other anti-inflammatory drugs [32], high concentrations of BHA also showed various cytotoxicity (Figure 2A). These could explain the influence of BHA concentration on its therapeutic effectiveness, suggesting that the dosage of BHA used for treating inflammation can be critically important. Thus, a lower dosage of BHA was used in subsequent in vivo studies.

The FCA-induced arthritis (AIA) rat model was established to evaluate the protective effects of BHA. AIA rat models have been commonly used to study the pathological mechanisms of immune-medicated diseases and also for preclinical studies of potential therapies [33]. In this study, AIA rats showed that symptoms were similar to those associated with RA. The histopathological assessment of the paw tissues revealed that an increased degree of inflammatory cell infiltration was encountered following the injection of FCA. Cytokine (TNFα, IL-1β and IL-6) levels and the expressions of p65 and p38 in the inflammatory joint tissues in AIA rats were significantly elevated in comparison with the control group, thus indicating that the AIA rat model was successfully established. BHA effectively alleviated the symptoms of paw and ankle swelling, significantly decreased paw thicknesses, ankle circumferences and arthritis scores. BHA reduced inflammatory cell infiltration and provided a better overall effect than the positive control drug DXMS, suggesting that BHA can be an alternative anti-inflammatory agent without the adverse effects caused by corticosteroid drugs.

Three major inflammatory signaling pathways including NF-κB, JAKs/STATs and MAPKs can cross talk with each other and play critical roles in triggering, promoting and regulating inflammatory responses. As shown in Figure 7, LPS induced the NF-κB signaling pathway and promoted the expression of cytokines, as well as increased cellular ROS level. Cytokines can induce the inflammatory responses through TNFR and cytokine receptors signaling pathways, which also lead to the production of inflammatory factors and the elevation of ROS. ROS can eventually trigger the activation of the NF-κB, JAK-STAT and MAPK [3,34] signaling pathways. The overexpression of TNFα is one of the major inflammatory events in RA, which contributes to the initiation of NF-κB and MAPK signaling pathways that induces the production of other inflammatory cytokines (i.e., IL-1β and IL-6) and metalloproteinases (MMPs). This study showed that BHA exerts its anti-inflammatory properties by decreasing the levels of TNFα, IL-1β and IL-6 in synovial tissues, thus showing potentials in preventing cartilage destruction and bone loss due to joint inflammation. Other studies also showed that blocking IL-1β and IL-6 pathways reduces joint damage and the clinical symptoms of RA [35,36]. Moreover, BHA provided a greater reduction in pro-inflammatory cytokine concentrations in joint tissues than was achieved with DXMS, further indicating the promising therapeutic properties of BHA.

Many signal transduction pathways are activated in the synovial inflammatory tissue of RA [37], and the NF-κB pathway appears to be a key regulator of inflammation in acute and chronic inflammatory diseases. The main activated form of NF-κB contains a trans-activating subunit of p65. We showed that BHA was more effective than DXMS in down-regulating the expression of p65. In addition to the NF-κB signaling pathway, the MAPK pathway also plays crucial roles in the immune-mediated inflammatory responses in RA [38]. The remarkable induction of p38 along with the increased expression of TNFα, IL-1β and IL-6 have been detected in the cultured fibroblast-like synoviocytes derived from RA patients [39]. We found that BHA can inhibit the expression of p38 in the AIA rat model, indicating that BHA can suppress p38/MAPK pathway. Since current clinical anti-inflammatory drugs such as DXMS and NSAIDs inhibit proliferation and induce apoptosis in cultured cells at lower concentrations than BHA [40,41], BHA could be a safer anti-inflammatory agent.

We have previously shown that BHA interacts with the TLR4 receptor on cell surfaces [18]. TLR4 plays the role by recognizing and binding to the stimuli to activate the inflammatory signaling pathway [26]. The action of BHA involves binding to TLR4 to prevent TLR4 from interacting with inflammation-inducing stimuli, thus regulating NF-κB and MAPK pathways. Herein, BHA interfered the expression of p65, p38 and STAT1/3, reducing the level of ROS, and the production of cytokines TNFα, IL-1β and IL-6 provided greater anti-inflammatory and antioxidative effects than DXMS. No adverse effects and body weight loss were observed in cell cultures treated with BHA at concentrations up to 0.1 mg/mL or among rats that had received this drug via injection, suggesting that BHA could provide a safer alternative to DXMS.

## 4. Materials and Methods

### 4.1. BHA Synthesis and Characterization

BHA was synthesized and characterized via the methods in our previous studies [18,19]. The obtained BHA was characterized via 1H NMR spectroscopy and matrix-assisted laser desorption ionization time-of-flight mass spectrometry (MALDI-TOF-MS) [19,42]. The percentage of *N*-butyryl substitution was determined based on the butylation-to-acetylation ratio of the BHA sample. The molecular weight of BHA was estimated via MALDI-TOF MS using the positive ion mode. 2,5-Dihydroxybenzoic acid (DHB) was used as a matrix.

### 4.2. Cell Culture

RAW264.7 cells (ATCC, Shanghai China) were cultured in Dulbecco’s Modified Eagle Medium (DMEM, Hyclone, Logan, UT, USA) supplemented with 10% fetal bovine serum (FBS, Hyclone, Shanghai, China), 100 IU/mL penicillin and 100 μg/mL streptomycin. Cells were incubated at 37 °C in a humidified atmosphere containing 5% CO_2_.

### 4.3. Cell Viability Assay

Cell viability was determined using MTT assays. Briefly, RAW264.7 cells were seeded into 96-well plates at a density of 8 × 10^3^ cells/well. After one night of incubation at 37 °C, a series of concentrations of BHA (0.01, 0.05, 0.1, 0.25, 0.5 and 1 mg/mL) was added into the corresponding wells and incubated for a further 6 and 24 h.

### 4.4. Immunofluorescence Assay

RAW264.7 cells were firstly seeded into 24 wells plate (5000 cells/well) and pre-treated with BHA at different concentrations for 12 h, followed by the addition of 1 µg/mL LPS and continue to grow for 12 h. Dexamethasone (DXMS) was used as a positive drug. After incubation, cells are washed with pre-cold PBST, fixed with 4% polyformaldehyde for 10 min, permeabilized with 0.25% Triton X-100, blocked with 2.5% bovine serum albumin in PBST for 1 h at room temperature and incubated with STAT1 and STAT3 primary antibodies (1:100 dilution with PBST) overnight at 4 °C. Cells were then incubated with a goat anti-rabbit IgG-conjugated FITC secondary antibody (1:200 dilution with PBST) for 1 h in dark at room temperature. Finally, the cells were stained at room temperature with 0.1 µg/mL DAPI for 1 min in dark. Images were captured using a Zeiss LSM 880 confocal microscope (magnification, ×400; Zeiss Microsystems GmbH, Oberkochen, Germany).

### 4.5. ROS Assay

The intracellular ROS concentration in RAW264.7 cells was determined with the use of carboxy-DCFH-DA as an oxidation-sensitive fluorescent dye. RAW264.7 cells were seeded at a density of 2 × 10^5^ cells/well in 12-well cell culture plates. After overnight incubation, the cell culture medium was replaced with fresh medium containing 1% FBS and BHA at various concentrations (0.05, 0.1 and 0.5 µg/mL) in the presence or absence of LPS (1 µg/mL) and incubated for 12 h. Reducing agent glutathione (GSH, 20 mM) were used as the positive control. DXMS (Shiyao Yinhu Pharmaceutical Co., Ltd., Shijiazhuang, China) were used for comparison purposes. 2,7-dichlorofluorescein diacetate (DCFH-DA) detection reagent measuring 10 mM was added into the wells after the washing steps. After incubation in darkness for a further 30 min at 37 °C, the cells were washed with PBS to completely remove the free DCFH-DA from the cells. Finally, DCF fluorescence distributions were analyzed via fluorescence microscope with excitation and emission wavelengths of 488 and 525 nm, respectively.

### 4.6. Adjuvant-Induced Arthritis (AIA) Rat Model

Male Sprague-Dawley (SD) rats were purchased from the Biological Products Institute (Changchun, China). SD rats were housed in plastic cages and maintained on a 12 h light/dark regimen (lights on 7:00–19:00 h) under standard laboratory conditions of 23 ± 1 °C with 55% relative humidity. SD rats were provided standard feed and tap water ad libitum. The protocols of the animal experiments were reviewed and approved by the Animal Ethics Committee of Jilin University (Reference No. 202009007).

SD rats (weighing ~180 g) were randomly divided into four groups (*n* = 6), including a control, model, positive drug DXMS and BHA groups. At day 0, 50 μL of sterilized saline solution was injected into the pad on the right paw of each rat in the control group, and the other four groups were administered with 50 μL of Freund’s Complete Adjuvant (FCA, Sigma, St. Louis, USA) via the same subcutaneous injection method to the right paw of each rat [43]. All of these rats were fed under standard conditions and the changes in the paws and ankles were observed and recorded.

### 4.7. Administration Method

The administration of DXMS or BHA began when the toe volume of the rats increased by 40% (at day 2). Sterilized saline solution (25 μL) was administrated by subcutaneous injection to the right hind paw of each rat in the control and model groups. The rats in the positive drug group were treated with 25 μL of DXMS (2 mg/mL), while 25 μL of BHA solution (1 mg/mL) was given to the rats in BHA group, respectively. The administrations were performed every second day for a total of 4 times at the right paws, as seen in Figure 4A.

### 4.8. Measurement of Paw Thickness and Ankle Circumferences

Along with the administration process, the paw thickness and the ankle circumference were recorded [44]. Body weights of the rats were recorded, and the paw shape in each group were photographed. After the administration period, the rats in each group were continuously observed for several days and then euthanized. The corresponding tissues and joints were collected and stored at −80 °C until use for the biochemical analysis.

### 4.9. Grade Scores Used for Rat Arthritis Assessment

The degrees of ankle inflammation and bone erosion were examined and assigned scores of 0–4 points [45]. The scoring criteria for the degree of inflammation were as follows: 0 = normal and no swelling; 1 = slight swelling of the rat soles; 2 = moderate swelling around the soles and joints; 3 = severe swelling of the soles with joint deformation; 4 = severe swelling with bone damage and local ulcers.

### 4.10. Enzyme-Linked Immunosorbent Assay

RAW264.7 cells were seeded into 96-well plates at a density of 2 × 10^4^ cells/well. After overnight incubation, the cell culture medium was replaced with fresh medium containing 1% FBS and BHA at various concentrations. DXMS was used as a positive control drug. After 6 h of incubation, the medium was replaced with fresh medium containing 5.5% FBS, and cells were stimulated with 1 µg/mL LPS for 12 h [46]. The culture medium supernatants were collected, and the protein levels of secreted TNFα, IL-1β and IL-6 were determined using enzyme-linked immunosorbent assay (ELISA) kits (Boster Biological Technology, Ltd., Wuhan, China) according to the manufacturer’s instructions.

The rats were euthanized at the end and joint tissues were collected to determine protein levels of TNFα, IL-1β and IL-6 with ELISA kits (purchased from CUSABIO, Wuhan, China), according to the manufacturer’s instructions.

### 4.11. Histopathological Assessment

The inflamed tissues between the joint and sole of the rats were fixed in 4% paraformaldehyde overnight, then embedded in paraffin and sectioned longitudinally. Serial 4 mm sections of the tissues were stained with hematoxylin and eosin (H&E) and assessed under a microscope (40× to 400×, final magnification). The histopathological changes exhibited by the paw tissue were assessed with regard to the degree of inflammatory cell infiltration.

### 4.12. Real-Time Quantitative PCR

Total RNA was isolated from tissues using Trizol reagent kit (Transgen, Beijing, China) according to the manufacturer’s protocol (*n* = 3 in each group). The purified RNA was reverse transcribed into complementary DNA (cDNA) with a mix of oligo dT and random primers (Transgen, China). The mRNA levels of p65 (NF-κB) and p38 (MAPK) were quantified by qPCR, as described [47]. The expression of target genes was normalized to the amount of housekeeping GAPDH transcripts in the same cDNA sample. The relative expression of target genes was determined using the 2^−∆∆CT^ method: where ∆CT_(Control)_ = CT_(Target gene)_ − CT_(GAPDH)_, ∆CT_(Test)_ = CT_(Target gene)_ − CT_(GAPDH)_ and ∆∆CT = ∆CT_(Test)_ − CT_(GAPDH)_, as described [48]. The mRNA expressions were normalized to the house keeping GAPDH and presented as percentages of the controls. The primer sequences were: p65 (forward: 5′-GCC TCA TCC ACA TGA ACTTGT GGG-3′; reverse: 5′-ACC ATG GTC TGG GCA AGG ACT GGG-3′), p38 (forward: 5′-AGCTGTCGAGACCGTTTCAG-3′; reverse: 5′-AGGTTGCTGGGCTTTAGGTC-3′ and GAPDH (forward: 5′-ACC ACA GTC CAT GCC ATC AC-3′; reverse: 5′-TCC ACC ACC CCC TGT TGC TGT A-3′).

### 4.13. Statistical Analysis

Data were represented as mean ± standard error of mean (SEM). A one-way analysis of variation (ANOVA) was used for comparison between multiple groups. A value of *p* < 0.05 was considered to be statistically significant.

## 5. Conclusions

The present study clearly shows that BHA exerted anti-inflammatory effects by inhibiting the production of pro-inflammatory cytokines TNF-α, IL-1β and IL-6 in both LPS-induced macrophages and in FCA-induced AIA rats by regulating NF-κB, JAK-STATs and MAPK inflammatory signaling pathways. BHA reduced the ROS levels in LPS-induced macrophage cells, which can play an active role in protecting articular tissues. BHA exhibited better anti-inflammatory and antioxidant effects in comparison to DXMS, indicating that BHA is a promising candidate for use as an anti-inflammatory agent.

## Data Availability

Not applicable.

## Supplementary Materials

The following supporting information can be downloaded at: https://www.mdpi.com/article/10.3390/molecules27103267/s1, Figure S1: Effects of BHA on the expression of TNFα in LPS-induced RAW264.7 macrophage cells. Figure S2: Effects of AHA on the ROS level in LPS-induced RAW264.7 macrophages.

## Abbreviations

HA, hyaluronic acid; BHA, *N*-butyrylated hyaluronic acid; TNFα, tumor necrosis factor α; IL-1β, interleukin-1β; IL-6, interleukin-6; ROS, reactive oxygen species; NF-κB, nuclear factor kappa-B; MAPK, mitogen-activated protein kinase; GSH, glutathione; FCA, Freund’s adjuvant; DXMS, dexamethasone; DCFH-DA, 2,7-dichlorofluorescein diacetate; AIA; adjuvant-induced arthritis.
