# Supplementary material for: N-Butyrylated Hyaluronic Acid Achieves Anti-Inflammatory Effects In Vitro and in Adjuvant-Induced Immune Activation in Rats
Source: Molecules. 2022 May 19;27(10):3267. doi: 10.3390/molecules27103267 (PMC9145605; doi:10.3390/molecules27103267)
Supplement: Supplementary file 1 [file molecules-27-03267-s001.zip › molecules-1725155-supplementary.pdf]

Supplementary information

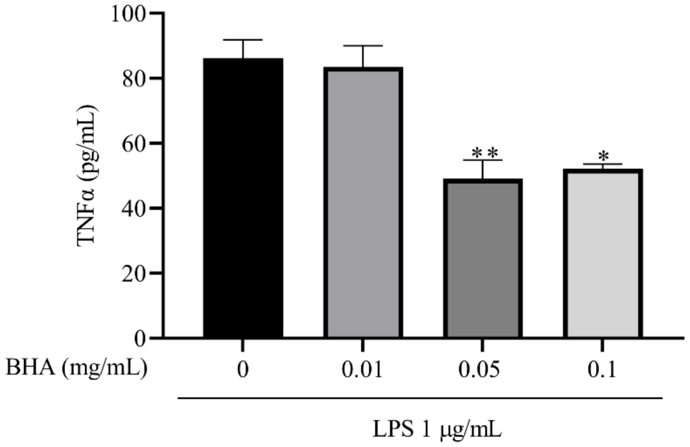

Figure S1. Effects of BHA on the expression of TNFα in LPS-induced RAW264.7 macrophage cells. Cells were pre-treated with various concentrations of BHA for 6 h and stimulated with 1 μg/mL of LPS for 12 h. Supernatants were collected after LPS stimulation and the protein level of TNFα was quantified by corresponding ELISA kits. Data are presented as the mean ± SEM of three independent experiments. One-way ANOVA was used to compare the results between each group. $^{*}p < 0.05$, $^{**}p < 0.01$.

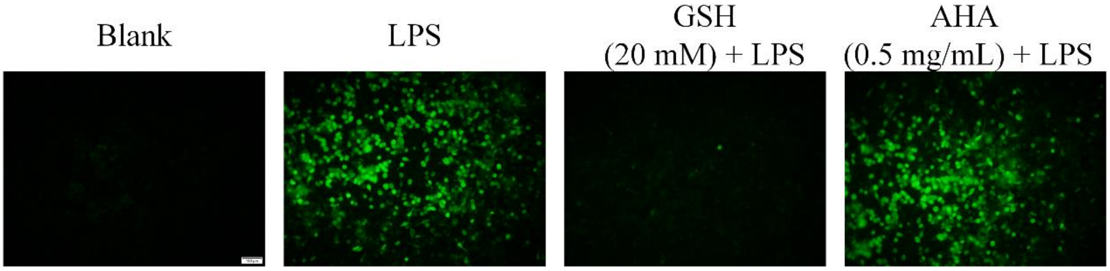

Figure S2. Effects of AHA on the ROS level in LPS-induced RAW264.7 macrophages. Cells were treated with AHA for 12 h in the presence or absence of LPS (1 μg/mL) for 12 h. GSH was used a control. The generation of ROS was measured and confirmed via DCFH-DA assays.
